# Epigenetic Regulation and Dietary Control of Triple Negative Breast Cancer

**DOI:** 10.3389/fnut.2020.00159

**Published:** 2020-09-08

**Authors:** Ornella I. Selmin, Micah G. Donovan, Barbara J. Stillwater, Leigh Neumayer, Donato F. Romagnolo

**Affiliations:** ^1^Department of Nutritional Sciences, The University of Arizona, Tucson, AZ, United States; ^2^University of Arizona Cancer Center, The University of Arizona, Tucson, AZ, United States; ^3^Department of Surgery, Breast Surgery Oncology, The University of Arizona, Tucson, AZ, United States

**Keywords:** triple negative breast cancer, epigenetic regulation, tumor suppressor, DNA methylation, dietary bioactive compounds

## Abstract

Triple negative breast cancer (TNBC) represents a highly heterogeneous group of breast cancers, lacking expression of the estrogen (ER) and progesterone (PR) receptors, and human epidermal growth factor receptor 2 (HER2). TNBC are characterized by a high level of mutation and metastasis, poor clinical outcomes and overall survival. Here, we review the epigenetic mechanisms of regulation involved in cell pathways disrupted in TNBC, with particular emphasis on dietary food components that may be exploited for the development of effective strategies for management of TNBC.

## Introduction

Breast cancer (BC) is the most common and deadly cancer in women worldwide, with a new case being diagnosed every 18 seconds (1, 2). Four molecular BC subtypes have been characterized, based on the expression of estrogen (ER) and progesterone (PR) receptor, and the human epidermal growth factor receptor-2 (HER2). The four molecular BC subtypes are usually classified as luminal ER positive (luminal A and Luminal B), her 2 enriched, and basal like [Table 1; (3)]. Luminal-A (LUM-A) are ER and/or PR positive, HER2 negative and have low proliferation markers such as Ki-67; luminal-B (LUM-B) are ER positive and/or PR positive, and either HER2 positive or negative with higher levels of Ki-67; her 2 enriched is ER and PR negative and HER2 positive; triple-negative/basal-like breast cancer (TNBC) is defined as ER and PR negative, and HER2-negative; LUM-A cancers are low-grade, tend to grow slowly and have the best prognosis, whereas LUM-B cancers generally grow slightly faster than LUM-A cancers and their prognosis is slightly worse. Her 2-enriched BC tend to grow faster than LUM-A and LUM-B tumors and can have a worse prognosis, but they are often treated with targeted therapies against HER2 with vastly improved outcomes (4). The TNBC subtype is more common in women with *BRCA1* mutations, among pre-menopausal, and African-American and Hispanic women (5).

**Table 1:** Molecular subtypes.

**Molecular subtype**	**ER**		**PR**		**Her 2**
Luminal A	Positive	And/or	Positive		Negative
Luminal B	Positive	And/or	Positive (or negative <20% and Ki67>14%)	or	Negative
Luminal B	Positive	And/or	Positive (or negative)		Positive
Her 2 enriched	Negative	and	Negative		Positive
Basal-like (TNBC)	Negative	and	Negative		Negative

Hope for successful treatment and prevention of BC was sparked by the identification in 1994 of the breast cancer 1 (*BRCA1*) gene (6). However, optimism was tempered by the finding that only a minor percentage (5–10%) of all BC associated with mutations in either BRCA1 or BRCA2. Nevertheless, carriers of mutated *BRCA* susceptibility genes have a higher risk of developing BC by age 70 (65% for mutated *BRCA1* and 45% for *BRCA2*), highlighting the important role of *BRCA* genes in BC development (7). Notably, TNBC are associated with mutations in the *BRCA* genes. In addition, sporadic, non-hereditary TNBC are often characterized by reduced or lost expression of BRCA1, also called BRCAness (8, 9). Increased *BRCA1* promoter methylation has been observed in a large number of TNBC (10), underscoring the importance of epigenetic factors contributing to the TNBC subtype.

The term epigenetics refers to external modifications that do not affect the DNA, but instead turn genes on or off through several mechanisms. Epigenetic mechanisms modulating gene expression include changes in DNA CpG methylation; histone post-translational modifications (e.g., methylation and acetylation), and expression of non-coding RNA. The study of factors, endogenous and exogenous, that modulate epigenetically the expression of genes involved in TNBC phenotype, is essential for the development of therapeutic strategies targeting TNBC. In this paper, we reviewed the mechanisms of action of endogenous factors and natural food components that modulate gene expression through epigenetic modifications, mainly DNA methylation and histone modifications, and identify possible targets for strategies of TNBC prevention or intervention.

## Source of Data

Research data published in English-language articles from the PubMed database were used for this review. Relevant studies were retrieved through the use of “triple negative breast cancer, epigenetics, dietary compounds” as keywords in searches of the database. The compounds analyzed in the second part of the review were chosen based on number of research articles found searching for “TNBC-Compound,” or “Breast Cancer-Epigenetic-Compound,” where “compound” was one of the following molecules: resveratrol, genistein, curcumin, (-)Epigallocatechin 3-gallate (EGCG), or folate. For each one of these compounds, between 18 and 40 research articles were found. In the “Other compounds” section we discussed bioactive molecules found searching for “TNBC-diet” and for which literature was less abundant.

## Triple Negative Breast Cancer

On average, only 15–20% of breast cancers are classified as TNBC, but have the poorest short and long-term prognosis (highest risk of local/regional recurrence, distant metastases, and cancer related mortality), largely due to lack of a targeted therapy (11). However, the percentage of TNBC varies by reproductive age being more prevalent in premenopausal women; BRCA1 mutation status; and in minority populations. For example, TNBC represent ~39% of all BC in African American women; ~ 20% in Hispanic White women; and ~16% in non-Hispanic/Caucasian White women of the same age (5). Over 80% of TNBC are invasive ductal carcinoma, with presence of lymphocytes, with a 4 to 6-fold increased risk of metastasis to the lung and the brain, rather than the bones (12). TNBC are highly heterogeneous and they have been classified in 6 distinct subtypes based on their gene expression: basal like (BL) 1 and BL 2, characterized by expression of genes involved in cell cycle and DNA damage, and high proliferative index; immunomodulatory (IM), expressing genes of the immune cell signaling pathways; mesenchymal (M) and mesenchymal stem-like (MSL) expressing genes involved in the epithelial-mesenchymal transition (EMT); and finally the LAR subtype positive for the luminal androgen receptor (AR), therefore responsive to therapy using AR antagonists such as bicalutamide (13). An alternative classification in four TNBC subgroups, combining BL1 with BL2, and M with MLS, was proposed by Burstein et al. (14) to account for tumor impurities derived by infiltrations of stromal and immune cells. Therefore, in addition to lacking target hormone receptors for targeted therapy (i.e., tamoxifen, herceptin, etc.), the heterogenicity within the TNBC subtype further complicates the design of effective neoadjuvant therapies.

Currently, a few treatment options exist for TNBC but have limited specificity. Taxanes are microtubules stabilizers that inhibit cell division. They have been shown to be more effective in the therapy of TNBC than in hormone receptor positive BC (15). Anthracyclines inhibit RNA synthesis and they have been used alone for the treatment of TNBC patients with limited success, but with better outcomes when used in combination with taxanes. Platinum agents induce cell death in BRCA1 mutant cells, due to their ability to prevent replication fork and inducing double strands breaks. Therapy with carboplatin and cisplatin (platinum compounds) was found to improve overall survival (27 vs. 8 months) in TNBC patients. The best clinical outcomes were achieved when platinum compounds were used in combination with other chemotherapeutic agents (9, 16). Epigenetic drugs, i.e., HDAC inhibitors (hydroxamic acids vorinostat, belinostat, LAQ824, panobinostat; and the benzamides: entinostat, tacedinaline, and mocetinostat) have been used in chromatin modifier therapies as adjuvants to sensitize TNBC cells. However, results have been mixed, as HDAC inhibitors induce re-expression of silenced tumor suppressors genes, but also inhibit expression of pro-apoptotic genes that down-regulate cell proliferation (17). Recently, a window-of-opportunity study examined the possibility of using valproic acid, a histone deacetylase inhibitor, as a possible neoadjuvant in TNBC patients. Even though a low number of women were available for evaluation, valproic acid treatment caused a 10% decrease in proliferation of breast tumors assessed by Ki-67 expression (18).

## Regulation of Tumor Suppressor Genes in TNBC

**Figure 1:**
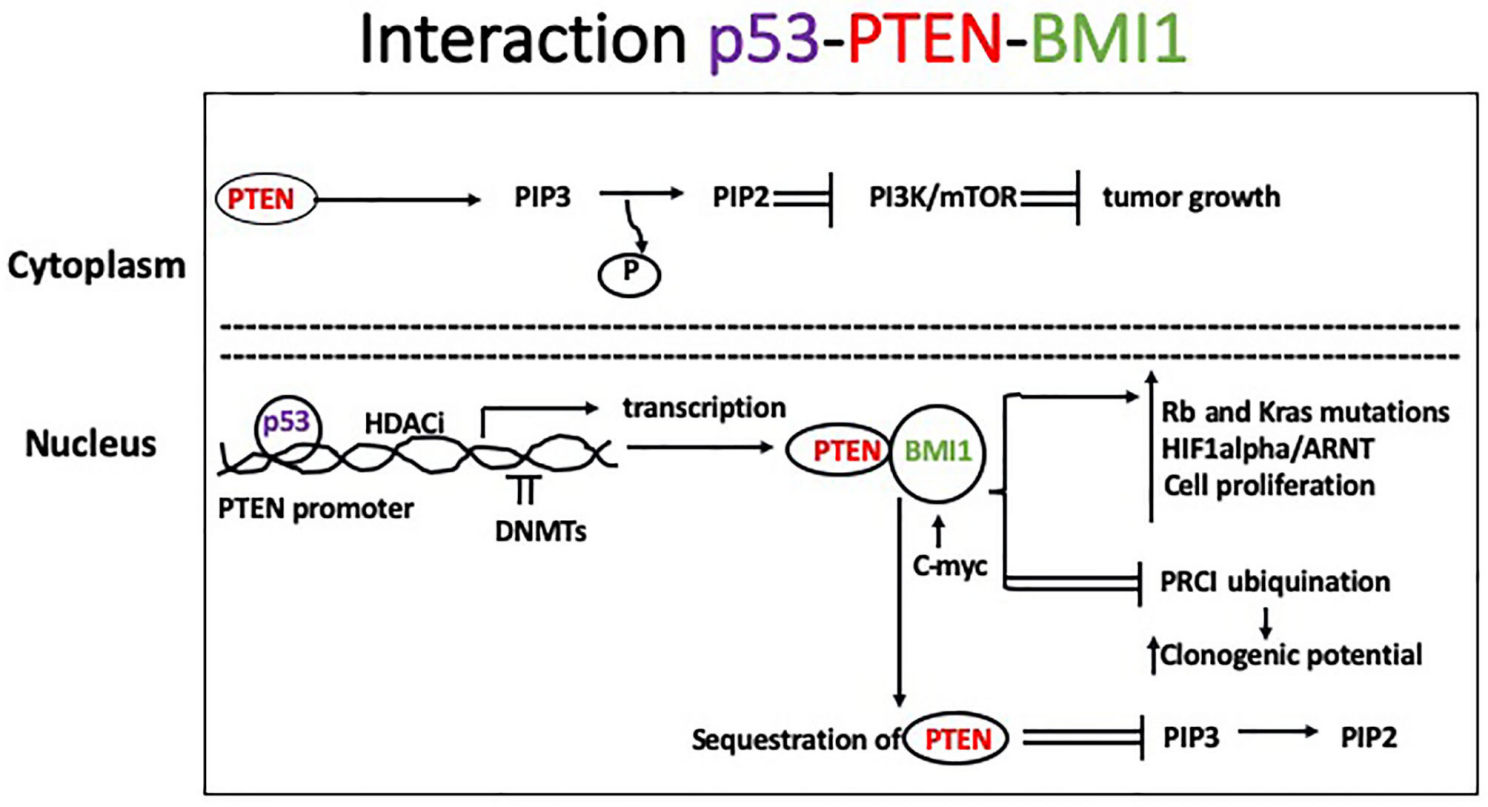
In the cytoplasm, PTEN mediates dephosphorylation of PIP3 to PIP 2 which inhibits the PI3k/Akt/mTOR signaling pathway leading to slower tumor growth. Histone deacetylase inhibitors activate PTEN nuclear translocation, whereas DNMTs cause hypermethylation of PTEN promoter and loss of its activity, which has been described during tumor progression. Binding of p53 to the *PTEN* promoter induces its transcription. Additionally, BMI1 binds to the PTEN protein in the nucleus inhibiting its expression. Consequently, BMI1 reduces PTEN's ability to inhibit Akt activation in the cytoplasm. Common features observed in BLBC include mutations of the tumor suppressor *Rb* and the oncogene *K-ras*, and increased activity of Myc and HIF1α)/ARNT, leading to increased cell proliferation.

Basal-like BC (BLBC) are mostly associated with BRCA1 mutations, but other tumor suppressor genes, such as *TP53* and *PTEN* (protein tyrosine phosphatase and tensin homolog), are often lost in this BC subtype. Somatic mutations of *TP53* are also found in the majority of TNBC. Reduced or lost activity of these genes is likely responsible for the high level of genomic instability observed in BLBC. PTEN is activated by phosphorylation at its K163 residue and translocates to the nucleus where it mediates DNA repair and chromosomal stability. In the cytoplasm, PTEN exerts lipid phosphatase activity by dephosphorylating phosphatidylinositol-3, 4, 5-triphosphate (PIP3) to PIP2 and inhibiting the PI3k/Akt/mTOR signaling pathway and tumor growth. Histone deacetylase inhibitors activate PTEN nuclear translocation, whereas hypermethylation of PTEN promoter and loss of its activity have been observed during BC progression. The PTEN promoter contains binding sites for p53, which induces PTEN transcription. B lymphoma Mo-MLV insertion region 1 homolog (BMI1) is a protein of the polycomb group involved in epigenetic regulation and overexpressed in many cancer types including breast (19). It has been implicated in promotion of anchorage-independent growth of tumor cells *in vitro* (20) and clonogenic potential by facilitating ubiquitination activity of protein regulator of cytokinesis 1 (PRC1). The PTEN protein binds in the nucleus to BMI1, and this interaction inhibits PTEN expression. Consequently, BMI1 reduces PTEN's ability to inhibit Akt activation, likely through its interaction with PTEN in the nucleus, making PTEN unavailable to dephosphorylate membrane-bound PIP3 to PIP2 (21). BMI1 also interacts directly with c-Myc, which binds to an enhancer sequence in the BMI1 promoter. Other common features observed in BLBC include mutations of the tumor suppressor Rb and the oncogene K-ras, as well as increased activity of Myc and hypoxia-inducible factor 1-α (HIF1α)/ARNT, indicating higher levels of cell proliferation (Figure 1).

Cancer cells metabolism is characterized by increased glucose uptake and lactate production (Warburg effect) (22). Pyruvate kinases are rate-limiting glycolytic enzymes involved in the final step of glycolysis (23). Two types of genes encode mammalian pyruvate kinase (pklr and pkm) (24). The PKM2 protein is regulated by several post-translational modifications that lead to the suppression of pyruvate kinase activity and translocation of PKM2 into the nucleus where it acts as a kinase toward specific nuclear proteins (25). It also acts as a co-activator of HIF-1α (26) and it contributes to tumorigenesis. It has been shown that PKM2 promotes angiogenesis through the activation of NF-κB/p65 and HIF-1α in diverse types of cancer (27, 28). The nuclear factor kappa light chain enhancer of activated B cells (NF-κB) family of transcription factors regulate inflammation, immune response, cell differentiation, proliferation, and survival (29), by forming protein complexes with DNA sequences at promoter regions of responsive genes. Specifically, NF-κB is frequently activated in TNBC and inhibition of NF-κB activity blocks growth of TNBC cells (30). Ma et al. (31) reported that knockdown of PKM resulted in anticancer effects against TNBC cells by reducing NF-κB activation and suggested it as a potential therapeutic strategy against TNBC cell growth. Other potential therapies leading to inactivation of NF-kB are currently being investigated (32).

## BRCA1 Function and Regulation

**Figure 2:**
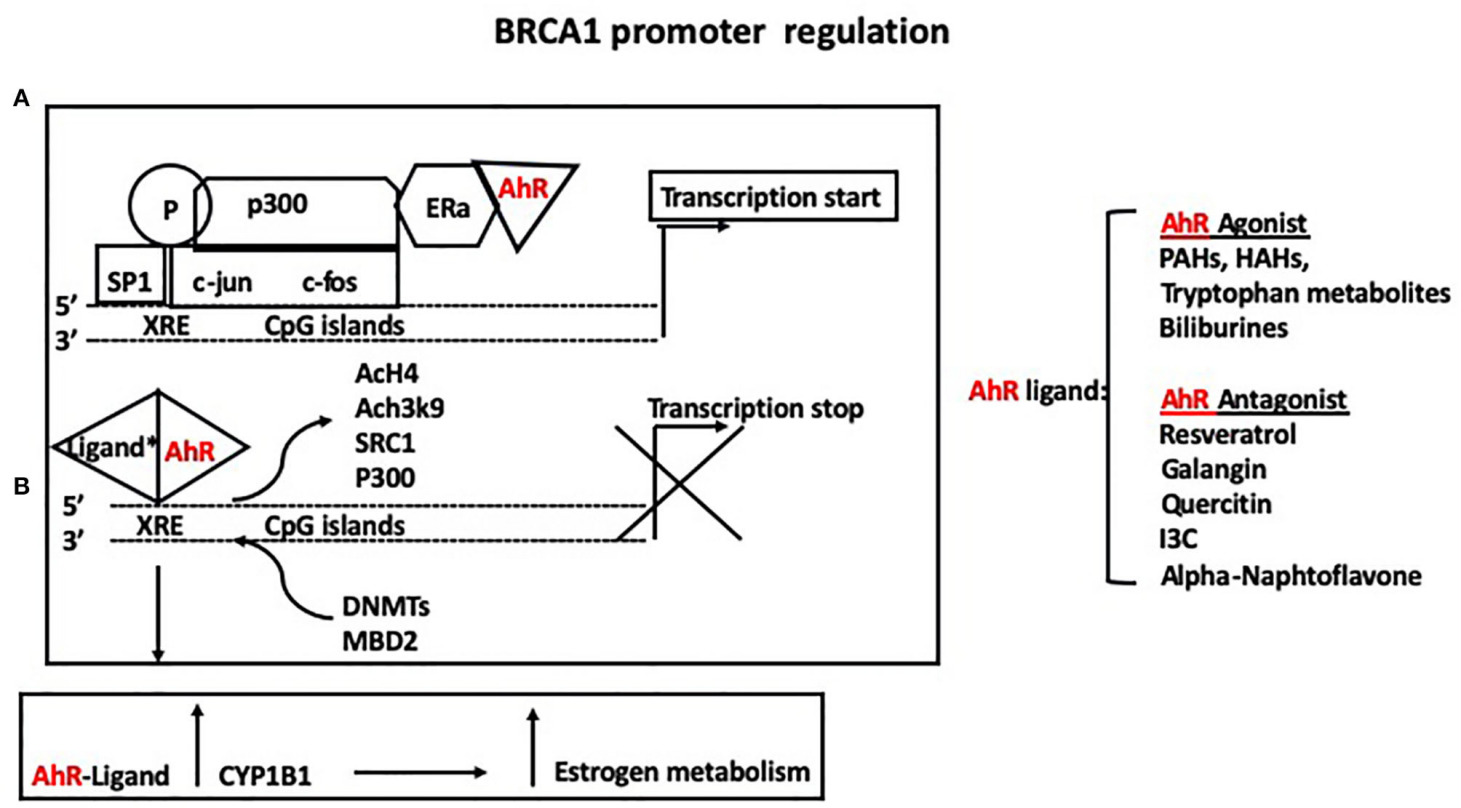
**(A)** The aromatic hydrocarbon receptor (AhR) binds directly to the *BRCA1* promoter and activates its expression in the presence of estrogen. **(B)** However, in the presence of either agonists or antagonists, the bound AhR is recruited to xenobiotic responsive elements (XRE = 5′-GCGTG-3′) harbored in the *BRCA1* promoter and inhibits its estrogen-mediated transactivation, by blocking the recruitment of ER, p300, SRC1, and histone modifications (i.e., AcH4, AcH3K9) that enhance transcription while promoting the association of factors (DNMTs, and methyl binding domain proteins such as MBD2) and histone modifications (H3K9me3, H3K27me3) that repress transcription. Binding of the xenobiotic TCDD activates AhR-mediated activity of P450 enzymes, including CYP1B1, which mediates estrogen metabolism.

The BRCA1 protein is a main player in DNA repair through homologous recombination and non-homologous end-joining (33–35). The loss of BRCA1 function is predominantly associated with the development of breast and ovarian cancer in women (35), and breast and prostate cancer in men (36). Our group summarized the role of nuclear receptors and other transcriptional factors in regulations of BRCA1 expression (37). In particular, activation of the ER by estrogen through association with p300, leads to BRCA1 basal transcriptional activation on the exon1b (38). In addition, binding of Sp1 to GC rich regions located upstream of an activator protein-1 (AP1) site in the *BRCA1* promoter, contributes to estrogen-dependent transcriptional activation (39). Phosphorylation and activation of the ER and Sp1 are also induced through non-genomic pathways involving a mitogen activated protein kinase (MAPK)-cascade, further regulating the cross-talk between ER and BRCA1. The aromatic hydrocarbon receptor (AhR) also binds directly to the *BRCA1* promoter and activates its expression in the presence of estrogen (40). However, in the presence of ligands, the bound AhR is recruited to xenobiotic responsive elements (XRE = 5′-GCGTG-3′) harbored in the *BRCA1* promoter and inhibits its estrogen-mediated transactivation, by blocking the recruitment of transcription factors and cofactors (ER, p300, SRC1) and histone modifications (i.e., AcH4, AcH3K9) that enhance transcription. Conversely, the bound AhR promotes the association of factors (DNMTs, and methyl binding domain proteins such as MBD2) and histone modifications (H3K9me3, H3K27me3) that repress transcription (Figure 2). The AhR and ER pathways also interact at multiple levels in a cell and tissue specific manner. For example, binding of the xenobiotic 2,3,7,8-tetrachlorodibenzo-p-dioxin (TCDD), activates AhR-mediated activity of P450 enzymes, including CYP1B1, which is involved in estrogen metabolism (41). Also, numerous exogenous and endogenous AhR ligands have been described, with different binding affinity for the AhR (42) and specificity for the two forms of the ER (α and β).

TNBC accumulate high levels of reactive oxygen species (ROS) because of their genetic (e.g., *BRCA1* silencing and *TP53* mutations) and metabolic alterations. Our group and others (43, 44) documented overexpression of AhR in TNBC, compared to other subtypes of BC. Specifically, Kubli et al. (44) reported that in normal and malignant mammary cells, AhR directly promoted the expression of amphiregulin (AREG), a ligand of the epidermal growth factor receptor (EGFR). The authors demonstrated that AhR–AREG signaling pathway induced tumorigenesis by controlling ROS and promoting the tumorigenic functions of the tumor microenvironment. They further showed that AhR loss of function sensitized tumor cells to Erlotinib, an EGFR inhibitor, suggesting a promising combinatorial antitumor strategy for the treatment of TNBC. Importantly, these data implicate a causative role of the AhR in the development of TNBC.

**Figure 3:**
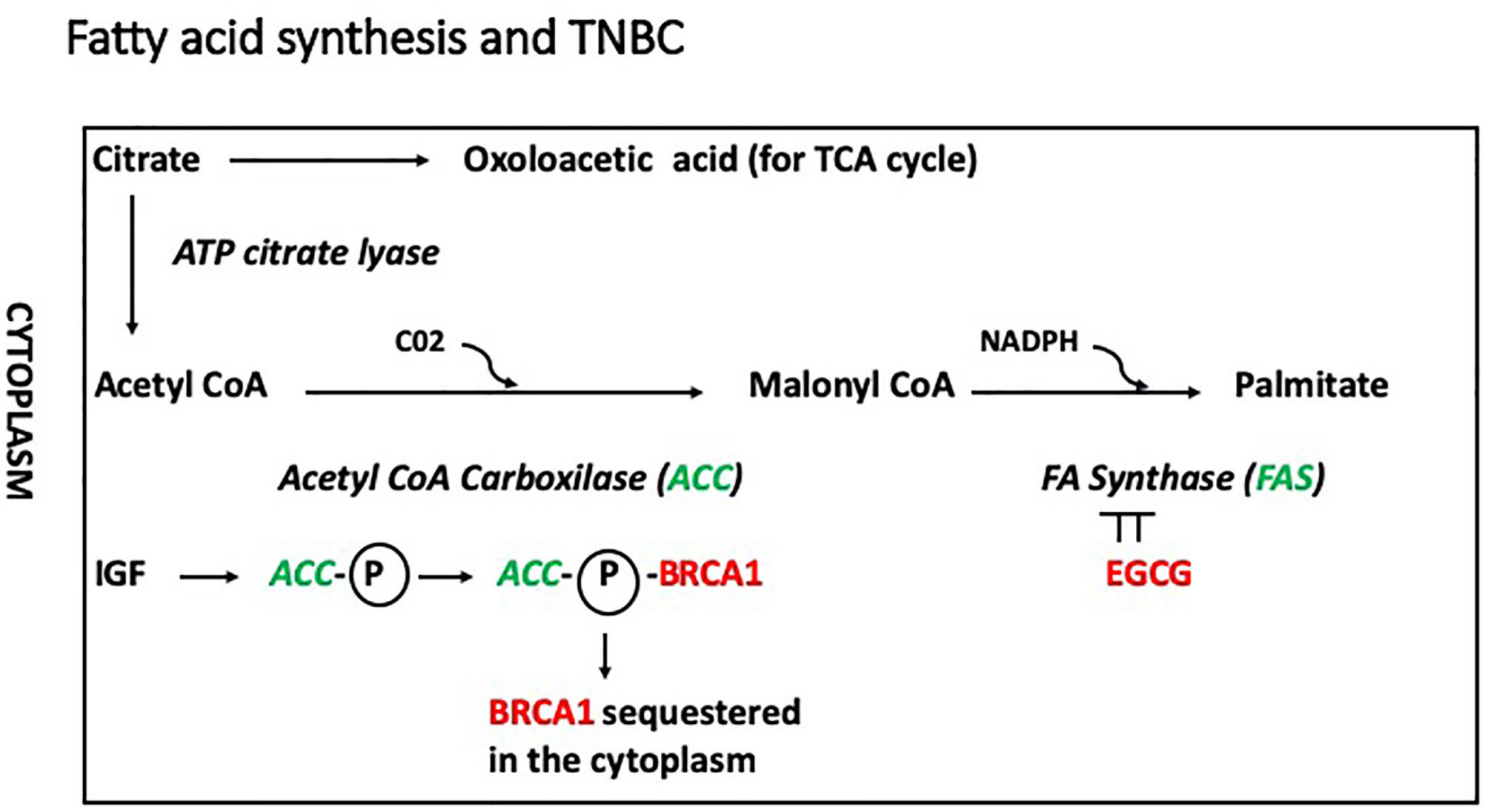
In this model, IGF-1 induces phosphorylation of ACCA, and inhibits its association with BRCA1, which remains inactive in the cytoplasm. In addition, phosphorylated ACC would promote fatty acid synthesis inducing conversion of acetyl coA into malonyl coA. The conversion of malonyl coA into palmitate is mediated by the fatty acid synthase (FASN), which is inhibited by epigallocatechin-3-gallate (EGCG).

Recently, a novel non-genomic role for BRCA1 has been described in which the BRCA1 protein in the cytoplasm interacts with acetyl CoA carboxylase (ACCA) in a phospho-dependent manner, and this interaction modulates lipogenesis (Figure 3). In this model, IGF-1 induces phosphorylation of ACCA, and inhibits its association with BRCA1 (45, 46). Cytoplasmic BRCA1 is rare in less aggressive ER-positive BC (47) and has been linked to metastasis in older patients (>40 years of age) (48). In ER positive cells, Koobotse et al. (46) concluded that inhibiting the association between cytoplasmic BRCA1 and ACCA, e.g., by increasing IGF-1 activity or reducing BRCA1 levels, would induce fatty acid synthesis and promote cell growth. Further studies are necessary to elucidate how the BRCA1/ACCA/IGF-1 axis contribute to tumorigenesis.

Because loss of BRCA1 function is commonly observed in sporadic BC and in subgroups of TNBC, there is great interest in food components that may increase the expression and function of the BRCA1 protein, for developing therapeutic strategies aimed at preventing or stopping BC development. Hypermethylation of the BRCA1 promoter gene has been described in 30–65% of TNBC, and some studies have suggested that *BRCA1* hypermethylation is a hallmark of this BC subtype (49, 50). Hypermethylation of *BRCA1* and *ESR1* (ERα) genes are usually concurrent (51).

## Bioactive Food Components

### Resveratrol

Resveratrol (3,4′,5-trihydroxystilbene) is a polyphenolic compound naturally occurring in the skin of dark colored fruits as grapes and berries, in peanuts and strongly pigmented vegetables. Certain plants produce resveratrol and other stilbenoids in response to stress, injury, fungal infection, or ultraviolet (UV) radiation. Resveratrol has been extensively studied *in vitro* and in animal models for its properties against cardiovascular and cognitive diseases, metabolic diseases as diabetes type 2, and cancer (52). Several studies have investigated the mechanisms of action of resveratrol in BC cells. Our group (53) reported that resveratrol effectively reversed several epigenetic changes associated with activation of the AhR and its binding to the BRCA1 promoter, in ER/PR positive breast cancer (MCF-7) cells. In particular, resveratrol (10 and 20 μM) antagonized the association of H3K9me3, DNMT1, and MBD2 on the *BRCA1* promoter; increased transcription of *BRCA1*; and reduced accumulation of DNA strand breaks. Recently, Chatterjee et al. (54) reported that resveratrol increased the expression of BRCA1, p53, and p21, while decreasing protein arginine methyltransferase5 (PRMT5) and enhancer of zeste homolog 2 (EZH2) in both MCF-7 and MDA-MB-231 (TNBC) cells. In particular, exposure to resveratrol induced loss of catalytic products of PRMT5, H4R3me2S, and H3K27me3. Moreover, resveratrol reduced lysine deacetylase (KDAC) activity and expression of KDAC1-3, whereas the expression of lysine acetyltransferase KAT2A/3B was enhanced. Overall, resveratrol increased H3K9ac and H3K27ac activity, while reducing repressive histone marks (H4R3me2s and H3K27me3) and increasing the level of activating histone marks (H3K9/27ac) in the proximal promoter region of the tumor suppressor genes *BRCA1, p53*, and *p21*. The authors observed that the effects of resveratrol were more pronounced in TNBC cells MDA-MB-231, compared to ER positive MCF7 cells. Other studies showed that resveratrol reduced the expression of metastasis associated protein 1 (MTA1), which is a component of the NuRD complex, functioning as a nucleosome remodeling and deacetylating unit. The resveratrol-induced disruption of the complex MTA1-HDAC lead to increased expression and acetylation of p53, BAX, p21, and apoptosis (55). Earlier studies had reported increased expression of PTEN induced by resveratrol in BC cells (56).

Lysine acetylation within the signal transducer and activator of transcription 3 (STAT3) can interfere with the interaction between DNMT1 and STAT3 causing demethylation and re-expression of tumor suppressor genes. In the TNBC cell line MDA-MB-468, treatment with resveratrol significantly reduced STAT3 acetylation as well as *ESR1* (ERα) gene promoter DNA methylation. This resulted in the increased expression of ERα and the sensitization to ER-targeted therapy with tamoxifen. Further, growth of *in vivo* tumors in mice was significantly reduced by resveratrol combined with tamoxifen, but not by tamoxifen alone (57).

We reported that the *in utero* exposure to TCDD increased the number of terminal end buds (TEB) (undifferentiated, proliferative structures) and reduced BRCA-1 expression in mammary tissue of rat offspring. The treatment with TCDD induced occupancy of the BRCA-1 promoter by DNMT-1, CpG methylation of the *BRCA-1* promoter, and expression of cyclin D1 and cyclin-dependent kinase-4 (CDK4). These changes were partially overridden by pre-exposure to resveratrol, which stimulated the expression of the AhR repressor AhRR, and its recruitment to the *BRCA-1* gene (58). In this study, the concentration of resveratrol in the prenatal diet (7 ppm) approximated the level (10 ppm) used in previous studies to prevent mammary carcinogenesis induced by agonists of the AhR (59). Taken together, these results suggest that the anti-DNMT properties of resveratrol may be a useful tool for prevention of BRCA-1-related tumors.

In a pilot study enrolling 39 women with increased BC risk, the subjects were divided into three groups, receiving 0, 5, and 50 mg resveratrol, twice daily for 3 months. An inverse relationship between serum resveratrol levels and RASSF-1α methylation was observed, leading to higher levels of expression of this tumor suppressor gene (60). One of the practical problems presented by resveratrol is how to reach effective *in vivo* concentrations, due to its poor water solubility. To overcome the low bioavailability of resveratrol, oxidized mesoporous carbon nanoparticles (OMCNs) with size below 200 nm and high solubility in water were used to encapsulate resveratrol for delivery in TNBC cells. The results showed good biocompatibility, and cellular uptake efficiency. *In vitro* toxicity and apoptosis analyses indicated that the effects of resveratrol were mediated by poly(ADP-ribose) polymerase (PARP) and caspase-3 cleavage (61).

In a recent article, Lucas et al. (62) suggested that resveratrol and its derivative piceatannol, co-administered with anti-PDL1 (Programmed death-ligand 1) immunotherapy may result in positive response and improved clinical outcome in cancer patients expressing low levels of PDL1. The authors reported that in a panel of BC cell lines, including the TNBC cell line Cal51, the expression of PD-L1 was up-regulated through HDAC3/p300-mediated NF-κB control.

Thus, hypothetically, even though the upregulation of PD-L1 by polyphenols in cancer could promote disease progression, agents capable of upregulating PD-L1 expression in tumor cells could sensitize cancer cells for an improved clinical response to PD-L1 immune checkpoint blockade therapy. The authors cautioned that the upregulation of PD-L1by resveratrol or piceatannol occurred at doses not achievable physiologically.

### Genistein

Genistein and daidzein are the most abundant isoflavones found in various legumes, grains, and vegetables, with soybeans contributing to the higher amount of isoflavones in the human diet (63). Average dietary isoflavone intakes in Asian countries range from 25 to 50 mg/day (64), in contrast, the levels are on average 10-fold lower in Western countries (65).

Consumption of high levels of soybeans associated with early and lifelong exposure to isoflavones have been suggested as possible reasons for the lower rate of BC in Asian populations. Epidemiological studies have shown that higher levels of dietary soy isoflavones (≥20 vs. ≤5 mg/day) were linked to a 29% reduced risk of BC in Asian and American Asian women (66), and early soy exposure may lower risk of BC later in life (67). Also, a meta-analysis of four prospective cohort studies indicated that high vs. low isoflavone intakes (>20 vs. <5 mg/day) might reduce risk of recurrence (RR = 0.84, 95% CI: 0.71–0.99) in BC survivors (68). An inverse association between soy isoflavone intake and recurrence was shown only among women undertaking tamoxifen treatment (69). Even if there are not sufficient data for discouraging moderate consumption of dietary isoflavones, it was recommended that women with a history of ER positive BC, should not increase their consumption of phytoestrogens, including soy isoflavones (70). Guo et al. (71) studied the effects of long-term pre-diagnosis soy food intake on the expression of 800 miRNAs and 302 pre-selected genes in tumor tissues from 272 TNBC cases from the Shanghai Breast Cancer Survival Study. Their findings indicated that soy food consumption (on an average of 10.8 grams/day) for a year before diagnosis may lead to increased expression of tumor suppressor genes and miRNAs, and decreased expression of oncogenes. In particular, higher soy food consumption was associated with higher levels of several miRNA involved in regulation of *TP53* cancer-related network, and lower expression of oncogenes such as *KRAS* and *FGFR4* (71).

Soy isoflavones have weak estrogenic activity due to their structural similarity with 17-β-estradiol, and they can act as estrogen agonists or antagonists based on their binding to either the ERα or ERβ, respectively. It has been shown *in vitro* that genistein inhibits protein tyrosine kinases (e.g., EGFR, PDGFR), NF-kB (72), and DNMT (73–76). In ERα positive BC cells, we reported (77) that genistein reversed hypermethylation of the *BRCA1* promoter in part through antagonism of constitutively active AhR. We recently extended our studies in TNBC cell lines showing that genistein upregulated *BRCA-1* expression leading to expression of ERα, and these effects were linked to acquired sensitivity of TNBC cells to the growth inhibitory effects of tamoxifen (78).

Other groups reported that the treatment of MDA-MB-231 (TNBC) cells with 0, 5, 10, or 20 μM genistein induced apoptosis and cell cycle arrest in G2/M in a dose-dependent manner. The authors suggested that these effects were mediated by inhibition of NF-κB activity via the Nocth-1 signaling pathway (79). Recently, Paul et al. (80) investigated the combinatorial effects of low doses of genistein and sulforaphane (SFN) on cell viability. SFN, which is enriched in cruciferous vegetables such as broccoli sprouts and kale, has been shown to possess HDAC inhibiting properties. The doses of 5 μM SFN + 10 μM genistein and 5 μM SFN + 15 μM genistein acted synergistically decreasing cellular viability in both ER positive and TNBC cell lines, and the activity of HDACs and HMTs. However, the combination of genistein and SFN was not effective in synergistically downregulating DNMT activity. Previous studies have shown that genistein increased acetylated histones 3, 4, and H3/K4 at the *p21* and *p16* transcription start sites, and also increased the expression of HAT enzymes that function in transcriptional activation (81). Thus, the combination of genistein and SFN acting synergistically may be due to the HAT promoting activity of genistein that activates tumor suppressor genes such as *p21* and *p16*, whereas SFN may be acting as an HDACi and suppressor of the activity of oncogenes.

Studies in rat and mouse models have produced inconsistent results regarding a protective effect of genistein on mammary tumorigenesis. In rats, mammary gland tumorigenesis was induced by exposure to 2,4-Dimethoxybenzaldehyde (DMBA), an AhR ligand, in animals receiving a wide range of genistein (from 10 to 300 ppm) at different stages of life. Data consistently showed that prepubertal exposure to genistein produced morphological changes in the mammary gland from a proliferative to a differentiated (reduced TEB) phenotype (82). Recently, Zhang et al. (83) reported that lifetime intake of genistein that mimicked Asian dietary patterns, reduced the *de novo* resistance to tamoxifen in rats, compared to a control group of animals that had received post-diagnosis genistein supplementation. The control group mimicked a dietary model in which genistein supplementation started in adulthood (Caucasian model).

In mice, most studies have been carried out in animals where mammary tumorigenesis was induced by an oncovirus, and genistein exposure was started in early life, pre, or post-puberty. Results of these studies were mixed and did not provide a clear answer whether genistein is protective against tumorigenesis. Possible reasons for this discrepancy include differences between species sensitivity to mammary tumors, tumor inducing agent (oncovirus in mice vs. tumor agent in rats), route, timing and dose of exposure to genistein [for a complete review Warri et al. (82)].

### Curcumin

Curcumin, extracted from the rhizome of turmeric and widely used in Indian medicine, shares with resveratrol biosynthesis pathways, short half-life, low retention, rapid elimination, and low availability of parent molecules. Piperine and β-glucan have been used as adjuvants for curcumin and resveratrol, respectively, and resulted in a substantial increase (2,000-fold for piperine) in their availability (84). In a recent study, Pandolfi et al. (85) reported on the development of a biomimetic nanodrug consisting of a self-assembling variant (HFn) of human apoferritin loaded with curcumin (CFn). The HFn construct improved the solubility, chemical stability, and bioavailability of curcumin, when tested in two cell lines (MDA-MB-468 and MDA-MB-231) representative of two TNBC subtypes. CFn enhanced the cytotoxic effect of doxorubicin, possibly by interfering with the activity of multidrug resistance transporters. In addition, CFn halted cell cycle in both cell lines, and inhibited Akt phosphorylation, suggesting that the effect on the proliferation and cell cycle were mediated by alteration of the PI3K/Akt pathway. In a study by Lv et al. (86), curcumin induced apoptosis in human BC cell line MDA-MB-231 (TNBC, basal-like). In a similar study, it was observed that curcumin inhibited the proliferation of MDA-MB-231 cells via the EGFR pathway (87). Fatty acid-binding protein 5 (FABP5), which has been indicated as a possible marker for interference with retinoic acid (RA) via the FABP5/PPAR β/δ pathway, was inhibited by curcumin and led to sensitization of the RA-resistant TNBC cells to RA-mediated growth suppression (88). Kundur et al. (89) reported that curcumin and quercetin (a flavonoid) exhibited synergetic anticancer effects in TNBC cells, possibly by promoting acetylation of the *BRCA1* promoter. Similarly to resveratrol, curcumin was found to stimulate β-oxidation of fatty acids, inhibiting adipogenesis, and inflammation (90). Al-Yousef et al. (91) recently reported that curcumin treatment restored *BRCA1* expression through reduction of its promoter methylation level in TNBC cell lines HCC-38 and UACC-3199. Lower levels of *BRCA1* promoter methylation were attributed to upregulation of the ten-eleven translocation 1 (TET1) gene, which mediates DNA demethylation via hydroxylation of 5-methylcytosine to 5-hydroxymethylcytosine. In addition, the authors suggested that TET1 may act as a target of miR-29b, an epi-miRNA which has been shown to inhibit both DNMTs and TETs (92). The miR-29b may balance DNA methylation levels, affecting the function of both methylation and demethylation enzymes (93).

### EGCG

(-)Epigallocatechin 3-gallate (EGCG), is the most abundant catechin found in green tea and displays strong antioxidant activity. Bao et al. (94) reported that exercise and consumption of green tea reduced recurrence and improved survival in TNBC patients. The antiproliferative effects of EGCG have been attributed to inhibition of fatty acid synthase (FASN), which is responsible for the *de novo* synthesis of palmitate, the most abundant fatty acid (Figure 3). Notably, FASN inhibition has negligible effect on non-malignant cells, which express low levels of FASN.

*In vivo*, EGCG displays low potency, poor bioavailability, and limited stability. Crous-Maso et al. (95) reported on the design and synthesis of a novel collection of polyphenolic compounds, containing two galloyl moieties (3,4,5-trihydroxybenzoyl group) linked by a variable cyclic subunit. These molecules were tested alongside EGCG for their anticancer properties, in particular in TNBC cell models.

It was reported that 92 % of tumor samples derived from 100 TNBC patients expressed FASN (96) and that doxorubicin-resistant cell lines were sensitive to chemotherapeutic drugs through inhibition of FASN (97). The same authors studied the effects of EGCG and its diester derivatives on a TNBC cell line, MDA-MB-231, and two models of the same cell line resistant to chemotherapy agents (98). Their results suggested that the highly proliferative phenotype of chemo-resistant TNBC cells could be treated with FASN inhibitors such EGCG or its more stable diesters.

Braicu et al. (99) evaluated the impact of *p53* silencing and EGCG treatment on genes involved in apoptosis in the Hs578T cell culture model of TNBC. The combined therapy led to the activation of pro-apoptotic genes (i.e., *Bcl-2*) and the inhibition of pro-survival genes (such as BAG cochaperone 3, X-linked inhibitor of apoptosis, and receptor interacting serine/threonine kinase 2), while reducing cell pathways leading to autophagy, thus confirming possible benefits of EGCG regimens for the prevention of TNBC.

In a recent study, Steed et al. (100) used suberoylanilide hydroxamic acid (SAHA), a HDAC inhibitor, alone or in combination with EGCG in the TNBC cell line MDA-MB-231. The two compounds (SAHA and EGCG) decreased the expression of cellular inhibitor of apoptosis 2 (*cIAP2*) while increasing the expression of pro-apoptotic caspase 7. The authors observed also changes in histone modifications, which may mediate the reduction in expression of *cIAP2*. Overall, these changes induced apoptosis. SAHA and EGCG further inhibited TNBC cell migration through fibronectin.

### Folate

Folates are water-soluble molecules functioning as methyl donors in one-carbon metabolism cycle, which requires vitamin B6, B12, and riboflavin. Fruits and dark leafy green vegetables are rich sources of folates (101). Folates are essential for the synthesis of amino acids and regulate the methylation of DNA and chromosomal stability by controlling the level of the methyl donor, S-adenosyl-methionine (*SAM*). Consequently, aberrant changes in folate metabolism may contribute to the development of cancer. As an example, *RAR B, BRCA1*, and Ras association domain family member 1 are frequently methylated in BC. Dietary intake of folate and cobalamin were found to be inversely associated with the methylation status of *RAR B* and *BRCA1* in a study by Pirouzpanah et al. (102). The same authors suggested that a low intake of folate and cobalamin correlated with the age-dependent tendency of promoter regions of these genes to be hypermethylated in tumors. On the other hand, increasing concentrations of folic acid were reported to cause a dose-dependent down-regulation of PTEN, APC, and RAR β2 tumor suppressor genes in both ER positive MCF-7 and triple negative MDA-MB-231 BC cell lines (103), suggesting caution with folic acid supplementation.

Folate receptor type alpha (FRA) is over-expressed by a majority of cancers including breast. Recently, two studies reported a strong association of FRA expression with ER/PR-negative and TNBC (>80%) status, poor prognosis, metastatic BC and worse overall/ disease-free survival (104, 105). Therefore, FRA represents a promising target against TNBC. In fact, some folate conjugates, such as folatefluorescein or folate-IgG showed promising anti-tumor activity in mice (106). Anti-FRA IgG antibodies such as MORAB-003 (Farletuzumab) have also been used to target ovarian cancer in patients, although with limited success (107).

In a recent study, Frontera et al. (108) demonstrated that an IgA Fc-folate conjugate can bind strongly to FRA receptors on TNBC stimulating neutrophils (PMN)-mediated cell killing. PMNs are heavily present in breast tumors and exert cytotoxic action against tumor cells, representing a possible tool against TNBC (109).

Cheung et al. (110) reported that TNBCs show dysregulated expression of thymidylate synthase, folate hydrolase 1, and methylenetetrahydrofolate reductase, involved in folate metabolism. This group used RNA interference to deplete FRα and showed a decrease in Src and ERK signaling which lead to reduced cell growth. An anti-FRα antibody (MOv18-IgG1) conjugated with a Src inhibitor was able to inhibit TNBC xenograft growth. Moreover, MOv18-IgG1 triggered immune-dependent cancer cell death *in vitro* by human volunteer and BC patient immune cells, and significantly restricted orthotopic and patient-derived xenograft growth.

Target genes and mechanisms of action of the main dietary compounds discussed in the manuscript are summarized in Table 2.

**Table 2:** Dietary compounds, their proposed target genes and mechanisms of action in *in vitro* or *in vivo* models of TNBC.

**Dietary compound**	**Targets**	**Epigenetic mechanism**
Resveratrol	BRCA1, p53, p21, BAX, PTEN, MTA1, ERa, RASSF-1a, PDL1	Promotes deacetylation/ demethylation through inhibition of H3K9me, DNMT1, MBD2, KDAC, and induction of H3K27ac and H3K29ac
Genistein	P53, KRAS-FGFR4, Tyrosine kinases, NF-kB, BRCA1, ERa, p21, p16	Acts as estrogen agonist or antagonist through binding to ER alpha or beta, respectively. Inhibits protein tyrosine kinases, DNMT; induces HAT activity
Curcumin	FAB P5/PPAR, BRCA1, FASN, miR-29b	Induces DNA acetylation, Beta-oxidation of FASN, inhibits promoter methylation
EGCG	FASN, Bcl2, cIAP2, Caspase7	Inhibits FA synthesis, HDAC
Folate	BRCA1, RARBeta, PTEN,	Modulates methylation through FRA, inhibits Src and ERK

### Other Phytochemicals

Chromatin modifier therapy is currently used as an adjuvant to sensitize TNBC cells with mixed results. HDAC inhibitors promote the re-expression of tumor suppressor silenced genes, and at the same time reduce the expression of pro-survival genes in favor of pro-apoptotic genes (17). Using phytochemicals to modify the methylation patterns of cancer cells and sensitize them to conventional treatments, may lead to a better alternative to general chemotherapy in particular for TNBC patients. To this end, Szarc Vel Szic et al. (111) showed that withaferin A (WA), a steroidal lactone, commonly known as Ashwagandha, Indian ginseng or Indian winter cherry, downregulated *HER2/PR/ESR*-dependent gene expression interactions and repressed aggressive triple-negative MDA-MB-231 BC cells with a specific DNA hypermethylation profile of tumor oncogenes. These included a urokinase-type plasminogen activator, ADAM metallopeptidase domain 8, tumor necrosis factor (ligand) superfamily member 12, and genes related to mitochondrial metabolism (malic enzyme 3, ME3) and enzymes of cell detoxification, such as glutathione S-transferase mu 1. In another study, physiologic concentrations of dietary phytochemicals, such as curcumin, DIM, EGCG, or indole-3-carbinol (I3C), altered DNA methylation and expression of genes involved in EMT (cadherin-11), p21Cip1, invasion (urokinase-type plasminogen activator), and interleukin-6. The authors concluded that, even though different targets, all these phytochemicals induced apoptosis of MDA-MB-231 cancer cells (112).

Li et al. (113) reported that fucoidan (a complex sulfated polysaccharide extracted from brown seaweed) inhibited migration of TNBC cells through reduced expression of markers of EMT (N-cadherin and vimentin). Also, the expression of *HIF1a*, which is elevated in metastatic cancer characterized by drug-resistance and high mortality rate, was reduced by fucoidan. However, HIF1a expression does not appear to be different in TNBC compared to other BC subtypes (114).

Inactivation of *TP53* is a requirement for tumor progression in BRCA1 deficient BC, and current therapies include treatment with HDAC inhibitors. Zinc metallochaperones are being developed as anticancer drugs that target a class of zinc-binding p53 mutations by restoring wildtype p53 structure and function (115). Consumption of pomegranate has been observed to reduce beta-catenin, EMT and overall metastasis in TNBC (116). In a recent study, Rzepecka-Stojko et al. (117) evaluated the *in vitro* cytotoxic activity of ethanol extract of propolis (EEP) and its derivative caffeic acid phenethyl ester (CAPE) toward the TNBC cell lines, MDA-MB-231 and Hs578T. The authors reported morphological changes of these cells were observed following exposure to EEP and CAPE. In addition, propolis and CAPE inhibited the growth of both cell lines in a dose-dependent and exposure time-dependent manner, with CAPE showing more cytotoxic activity than EEP. Recently, the anti-neoplastic effects of the electrophilic fatty acid nitroalkene derivative, 10-nitro-octadec-9-enoic acid (nitro-oleic acid, NO2-OA), were investigated in multiple preclinical models of TNBC (118). Electrophilic fatty acid nitroalkene derivatives (NO2-FA) are formed by the acidic conditions of digestion and the redox environment that is up-regulated during inflammation. Other electrophilic species present in vegetables such as broccoli, namely the isothiocyanate derivative sulforaphane, have been shown to mediate therapeutic actions in preclinical models of BC (119). The authors reported that NO2-OA reduced TNBC cell growth and viability *in vitro*, attenuated TNFα-induced TNBC cell migration and invasion, and inhibited the tumor growth of MDA-MB-231 TNBC cell xenografts in the mammary fat pads of female nude mice. These effects were mediated in part by inhibition of TNFα-induced NF-kB transcriptional activity and suppression of downstream NF-kB target gene expression, including the metastasis-related proteins intercellular adhesion molecule-1 and urokinase-type plasminogen activator.

## Conclusions

TNBC are heterogeneous in nature, highly metastatic, and their lack of steroid hormone receptors makes ineffective strategies targeting the ER (e.g., tamoxifen). The processes responsible for their propensity to metastasize primarily in lungs and brain are largely unknown, and contribute to the poor prognosis and high mortality rate in TNBC patients (120).

In this review, we underlined how transcription factors can alter the function of tumor suppressors involved in DNA damage, cell proliferation, and differentiation. Promoter methylation and histone deacetylation emerge as central epigenetic mechanisms silencing the expression of tumor suppressors, although the importance of other mechanisms (e.g., siRNA) has been amply described, in particular regarding the mode of action of dietary anticancer molecules (121). We highlighted the complexity of the systems that modulate the expression of *BRCA1*, whose absence of function is a characteristic of TNBC. Interestingly, the sequestration of BRCA1 in the cytoplasm through its interaction with the ACC enzyme provides a link between dysregulation of lipid metabolism and BRCA1 function in the development of TNBC. Investigations on fatty acid synthesis and their regulation are essential for understanding TNBC development and for identification of targets of treatment connected with inflammation and metabolic pathways, including arachidonic acid and prostaglandins (122). Of particular interest is the central role of the AhR, both as a modulator of BRCA1 function and as a possible target for development of therapeutic strategies against TNBC.

Numerous phytochemicals are being considered as promising allies in preventing or reversing different phases of TNBC progression *in vitro* and *in vivo*, and we described some of the more recent findings. One of the major challenges emerged from these studies is in general the poor bioavailability of these molecules, specifically their high degradability, low solubility, and high degree of metabolic transformation. Another critical issue is the lack of knowledge of how different phytochemicals interact with each other, and how their properties may be used for enhanced efficacy or to avoid negative interference.

Further studies are warranted to unravel the most promising strategies for the treatment of TNBC, through development of both novel drugs and use of available biomolecules. Implementation of dietary regimens rich in phytochemicals with anticancer properties and low in proinflammatory molecules also may prove to be a powerful tool for prevention and treatment of TNBC.

## Author Contributions

OS, MD, BS, LN, and DR contributed to the conception and development of the manuscript. OS and DR have primary responsibility for the writing of the manuscript. MD contributed to the writing of data related to BRCA1, AhR and genistein, and review and editing of the manuscript. BS and LN were responsible for the clinical content of the work and contributed to the writing and review of the manuscript. All authors contributed to the article and approved the submitted version.

## Conflict of Interest

The authors declare that the research was conducted in the absence of any commercial or financial relationships that could be construed as a potential conflict of interest.

## Abbreviations

ACCA: acetyl CoA carboxylase
AP-1: activator protein-1
AhR: aromatic hydrocarbon receptor
APC: Adenomatous polyposis coli gene
AREG: amphiregulin
ARNT: Aromatic hydrocarbon nuclear translocator
BC: Breast Cancer
BLBC: basal-like BC
BMI1: B lymphoma Mo-MLV insertion region 1 homolog
BRCA1: breast cancer protein 1
CDK4: cyclin-dependent kinase-4
CYP1B1: P450 cytochrome 1B1
DIM: diindolyl-methane
DMBA: 2,4-dimethoxybenzaldehyde
DNMT: DNA methyl transferase
EGCG: (-)epigallocatechin 3-gallate
EMT: epithelial mesenchymal transition
ERK: Extracellular Signal-Regulated Kinase
EZH2: enhancer of zeste homolog 2
ER: estrogen receptor
FABP5: fatty acid-binding protein 5
FASN: fatty acid synthase
FRA: folate receptor type alpha
HAT: histone acetylase transferase
HDAC: histone deacetylase
Her2: human epidermal growth factor receptor 2
HIF1: hypoxia-inducible factor 1
HMT: histone methyl transferase
KDAC: lysine deacetylase
KAT: lysine acetyltransferase
EGFR: epidermal growth factor receptor
FGFR4: fibroblast growth factor receptor 4
IGF-1: insulin growth factor-1
LUM-A/B: ER positive luminal A/B breast cancer
MAPK: mitogen activated protein kinase
MBD: methyl binding domain protein
MTA1: metastasis associated protein 1
NF-kB: nuclear factor kappa light chain enhancer of activated B cells
NuRD: nucleosome remodeling and deacetylating unit
PARP: poly(ADP-ribose) polymerase
PDL-1: programmed death-ligand 1
PI3k/Akt/mTOR: phosphatidylinositol-3-kinase/mammalian target of rapamycin
PIP3: phosphatidylinositol-3, 4, 5-triphosphate
PKM: mammalian pyruvate kinase
PR: Progesterone Receptor
PRMT5: protein arginine methyltransferase5
PRC1: protein regulator of cytokinesis 1
PTEN: protein tyrosine phosphatase and tensin homolog
RAR: Retinoic acid receptor
RASSF-1: Ras Association Domain Family Member 1
ROS: reactive oxygen species
SAM: S-adenosyl-methionine
STAT3: signal transducer and activator of transcription 3
TCDD: 2,3,7,8-tetrachlorodibenzo-p-dioxin
TEB: terminal end buds
TET 1: ten-eleven translocation 1
TNBC: Triple negative breast cancer
XRE: xenobiotic responsive elements.
